# 3-Hydroxy-2-[(2-hydroxy-4,4-dimethyl-6-oxocyclohex-1-en-1-yl)(thiophen-3-yl)methyl]-5,5-dimethylcyclohex-2-en-1-one

**DOI:** 10.1107/S160053681102407X

**Published:** 2011-06-25

**Authors:** Mohammad Asad, Chuan-Wei Oo, Hasnah Osman, Mohd Mustaqim Rosli, Hoong-Kun Fun

**Affiliations:** a School of Chemical Sciences, Universiti Sains Malaysia, 11800 USM, Penang, Malaysia; b X-ray Crystallography Unit, School of Physics, Universiti Sains Malaysia, 11800 USM, Penang, Malaysia

## Abstract

The asymmetric unit of the title compound, C_21_H_26_O_4_S, consists of two independent mol­ecules. In both mol­ecules, intra­molecular O—H⋯O hydrogen bonds stabilize the mol­ecular structure. In the crystal, each mol­ecule and its symmetry-related mol­ecule by twofold rotation form a dimer through a pair of inter­molecular C—H⋯O hydrogen bonds. In one of the mol­ecules, the thio­phene group is disordered over two sets of sites with occupancies of 0.735 (3) and 0.265 (3).

## Related literature

For general background to the title compound, see: Tietze & Beifuss (1991 ▶); Suh *et al.* (2000 ▶); Choudhary *et al.* (2006 ▶). For stability of the temperature controller used in the data collection, see: Cosier & Glazer (1986 ▶).
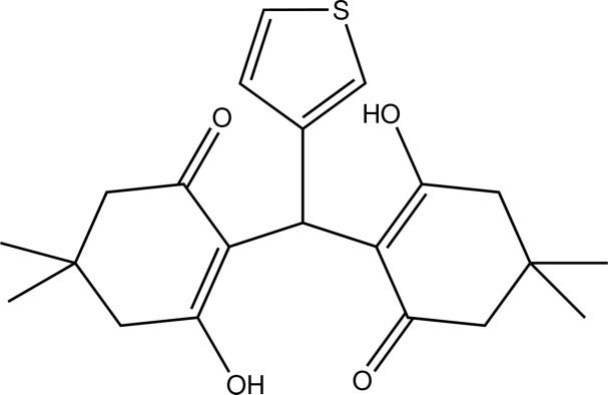


## Experimental

### 

#### Crystal data

C_21_H_26_O_4_S
*M* *_r_* = 374.48
Tetragonal, 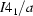

*a* = 20.3770 (1) Å
*c* = 36.9066 (7) Å
*V* = 15324.4 (3) Å^3^
*Z* = 32
Mo *K*α radiation
μ = 0.19 mm^−1^
*T* = 100 K
0.38 × 0.28 × 0.24 mm

#### Data collection

Bruker SMART APEXII CCD area-detector diffractometer
Absorption correction: multi-scan (*SADABS*; Bruker, 2009 ▶) *T*_min_ = 0.930, *T*_max_ = 0.955
162998 measured reflections
11335 independent reflections
9435 reflections with *I* > 2σ(*I*)
*R*_int_ = 0.053

#### Refinement

*R*[*F* ^2^ > 2σ(*F* ^2^)] = 0.049
*wR*(*F* ^2^) = 0.132
*S* = 1.05
11335 reflections
518 parameters
86 restraints
H atoms treated by a mixture of independent and constrained refinement
Δρ_max_ = 0.85 e Å^−3^
Δρ_min_ = −0.81 e Å^−3^

### 

Data collection: *APEX2* (Bruker, 2009 ▶); cell refinement: *SAINT* (Bruker, 2009 ▶); data reduction: *SAINT*; program(s) used to solve structure: *SHELXTL* (Sheldrick, 2008 ▶); program(s) used to refine structure: *SHELXTL*; molecular graphics: *SHELXTL*; software used to prepare material for publication: *SHELXTL* and *PLATON* (Spek, 2009 ▶).

## Supplementary Material

Crystal structure: contains datablock(s) global, I. DOI: 10.1107/S160053681102407X/is2734sup1.cif

Structure factors: contains datablock(s) I. DOI: 10.1107/S160053681102407X/is2734Isup2.hkl

Supplementary material file. DOI: 10.1107/S160053681102407X/is2734Isup3.cml

Additional supplementary materials:  crystallographic information; 3D view; checkCIF report

## Figures and Tables

**Table 1:** Hydrogen-bond geometry (Å, °)

*D*—H⋯*A*	*D*—H	H⋯*A*	*D*⋯*A*	*D*—H⋯*A*
O3*A*—H1*A*3⋯O1*A*	0.88 (3)	1.71 (3)	2.5638 (15)	164 (2)
O2*B*—H1*B*2⋯O4*B*	0.90 (3)	1.71 (3)	2.5719 (15)	159 (3)
O3*B*—H1*B*3⋯O1*B*	0.82 (3)	1.84 (3)	2.6569 (17)	170 (3)
O2*A*—H1*A*2⋯O4*A*	0.87 (3)	1.79 (3)	2.6393 (15)	165 (2)
C10*A*—H10*A*⋯O1*A*^i^	0.99	2.48	3.4530 (18)	169
C16*B*—H16*D*⋯O4*B*^ii^	0.99	2.60	3.5688 (18)	167

Symmetry codes: (i) 
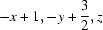
; (ii) 
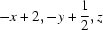
.

**Fig. 1.**
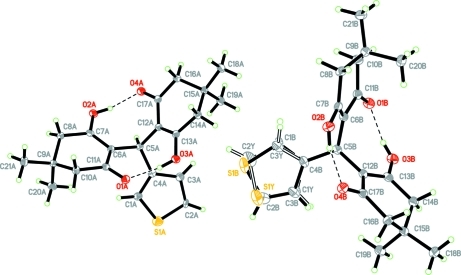
The molecular structure of the title compound, showing 50% probability displacement ellipsoids and the atom-numbering scheme. Hydrogen atoms are shown as spheres of arbitrary radius. Dashed lines indicate the O—H···O hydrogen bonds.

**Fig. 2.**
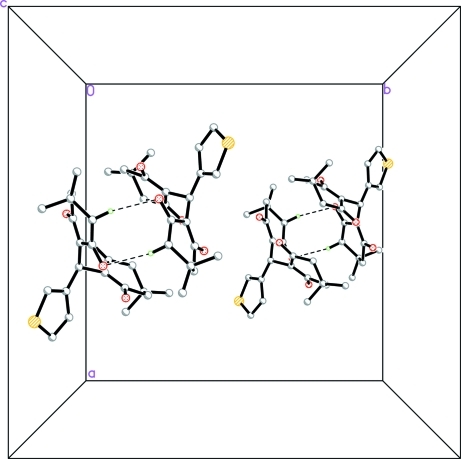
A packing diagram of the title compound, viewed along the c axis. Dashed lines indicate hydrogen bonds. Minor components and H atoms not involved in the hydrogen bonds have been omitted for clarity.

## supplementary crystallographic information

### Comment

We reported here the crystal structure of a bis compound which was synthesized from thiophene-3-carboxaldehyde and an active methylene compound, 5,5-dimethylcyclohexan-1,3-dione (dimedone) by using well-known Knoevenagel condensation reaction. This reaction was generally used in organic synthesis for C-C bond formation (Tietze & Beifuss, 1991) and active methylene compounds like dimedone was used as a precursor for many heterocyclic compounds (Suh *et al.*, 2000; Choudhary *et al.*, 2006).

There are two independent molecules in the the asymmetric unit (Fig. 1) and all parameters are within normal ranges. In one of the molecules, the thiophene group was disordered over two positions with refined site occupancies of 0.735 (3):0.265 (3). In both molecules, intramolecular hydrogen bonds O3A/B—H1A/B3···O1A/B and O2A/B—H1A/B2···O4A/B (Table 1) help  to stabilize the molecular structure. In the crystal structure, the two sets of molecules are interconnected by two intermolecular C10A—H10A···O1A^i^ and C16B—H16D···O4B^ii^ hydrogen bonds to form dimers (Fig. 2).

### Experimental

A mixture of thiophene-3-carboxaldehyde (4.46 mmol, 0.5 g) and dimedone (8.92 mmol, 1.25 g) in 20 ml of methanol was stirred overnight at room temperature. The completion of the reaction was monitored by TLC. After the reaction was completed, the crude product was separated, filtered, washed with methanol and dried. The isolated product was further purified by recrystallization from chloroform-methanol (1:1 *v*/*v*) to give the pure title compound in 85% yield.

### Refinement

One of the thiophene group was disordered over two positions with refined site occupancies of 0.735 (3):0.265 (3). The same *U*_ij_ parameters were applied to the C1B/C1Y and C3B/C3Y. Distance and rigid body restraint were used on disorder part of the molecular structure. O-bound H atoms were located from the difference map and refined freely. The rest of H atom were positioned geometrically with C—H = 0.95–1.00 Å and refined using a riding model, with *U*_iso_(H) = 1.2*U*_eq_(C) or 1.5*U*_eq_(C_methyl_).

### Crystal data

**Table:**

C_21_H_26_O_4_S	*D*_x_ = 1.298 Mg m^−^^3^
*M**_r_* = 374.48	Mo *K*α radiation, λ = 0.71073 Å
Tetragonal, *I*4_1_/*a*	Cell parameters from 9719 reflections
Hall symbol: -I 4ad	θ = 2.2–29.9°
*a* = 20.3770 (1) Å	µ = 0.19 mm^−^^1^
*c* = 36.9066 (7) Å	*T* = 100 K
*V* = 15324.4 (3) Å^3^	Block, colourless
*Z* = 32	0.38 × 0.28 × 0.24 mm
*F*(000) = 6400	

### Data collection

**Table:**

Bruker SMART APEXII CCD area-detector diffractometer	11335 independent reflections
Radiation source: fine-focus sealed tube	9435 reflections with *I* > 2σ(*I*)
graphite	*R*_int_ = 0.053
φ and ω scans	θ_max_ = 30.2°, θ_min_ = 1.8°
Absorption correction: multi-scan (*SADABS*; Bruker, 2009)	*h* = −28→28
*T*_min_ = 0.930, *T*_max_ = 0.955	*k* = −28→28
162998 measured reflections	*l* = −52→51

### Refinement

**Table:**

Refinement on *F*^2^	Primary atom site location: structure-invariant direct methods
Least-squares matrix: full	Secondary atom site location: difference Fourier map
*R*[*F*^2^ > 2σ(*F*^2^)] = 0.049	Hydrogen site location: inferred from neighbouring sites
*wR*(*F*^2^) = 0.132	H atoms treated by a mixture of independent and constrained refinement
*S* = 1.05	*w* = 1/[σ^2^(*F*_o_^2^) + (0.0603*P*)^2^ + 23.1894*P*] where *P* = (*F*_o_^2^ + 2*F*_c_^2^)/3
11335 reflections	(Δ/σ)_max_ = 0.001
518 parameters	Δρ_max_ = 0.85 e Å^−^^3^
86 restraints	Δρ_min_ = −0.81 e Å^−^^3^

### Special details

**Table:**

Experimental. The crystal was placed in the cold stream of an Oxford Cryosystems Cobra open-flow nitrogen cryostat (Cosier & Glazer, 1986) operating at 100.0 (1) K.
Geometry. All esds (except the esd in the dihedral angle between two l.s. planes) are estimated using the full covariance matrix. The cell esds are taken into account individually in the estimation of esds in distances, angles and torsion angles; correlations between esds in cell parameters are only used when they are defined by crystal symmetry. An approximate (isotropic) treatment of cell esds is used for estimating esds involving l.s. planes.
Refinement. Refinement of F^2^ against ALL reflections. The weighted R-factor wR and goodness of fit S are based on F^2^, conventional R-factors R are based on F, with F set to zero for negative F^2^. The threshold expression of F^2^ > 2sigma(F^2^) is used only for calculating R-factors(gt) etc. and is not relevant to the choice of reflections for refinement. R-factors based on F^2^ are statistically about twice as large as those based on F, and R- factors based on ALL data will be even larger.

### Fractional atomic coordinates and isotropic or equivalent isotropic displacement parameters (Å^2^)

**Table:**

	*x*	*y*	*z*	*U*_iso_*/*U*_eq_	Occ. (<1)
S1A	0.26498 (2)	0.53295 (2)	0.064534 (12)	0.02667 (10)	
O1A	0.43198 (5)	0.66891 (5)	0.06947 (3)	0.01577 (19)	
O2A	0.32661 (5)	0.79671 (5)	−0.01962 (3)	0.0185 (2)	
O3A	0.48148 (5)	0.58783 (5)	0.02392 (3)	0.0160 (2)	
O4A	0.38506 (5)	0.71916 (5)	−0.06694 (3)	0.0177 (2)	
C1A	0.29441 (7)	0.60778 (7)	0.05155 (4)	0.0174 (3)	
H1AA	0.2849	0.6476	0.0639	0.021*	
C2A	0.30093 (7)	0.49167 (6)	0.03039 (4)	0.0136 (2)	
H2AA	0.2976	0.4458	0.0261	0.016*	
C3A	0.33798 (7)	0.53824 (7)	0.00845 (4)	0.0184 (3)	
H3AA	0.3627	0.5260	−0.0123	0.022*	
C4A	0.33333 (6)	0.60401 (7)	0.02128 (4)	0.0140 (2)	
C5A	0.36197 (6)	0.66325 (6)	0.00161 (3)	0.0126 (2)	
H5AA	0.3278	0.6755	−0.0166	0.015*	
C6A	0.36899 (6)	0.72403 (6)	0.02540 (4)	0.0126 (2)	
C7A	0.34849 (7)	0.78460 (7)	0.01386 (4)	0.0143 (2)	
C8A	0.34971 (7)	0.84505 (7)	0.03702 (4)	0.0168 (3)	
H8AA	0.3092	0.8706	0.0324	0.020*	
H8AB	0.3875	0.8725	0.0296	0.020*	
C9A	0.35486 (7)	0.83136 (7)	0.07775 (4)	0.0139 (2)	
C10A	0.40850 (7)	0.77994 (7)	0.08357 (4)	0.0154 (3)	
H10A	0.4517	0.8009	0.0794	0.018*	
H10B	0.4072	0.7655	0.1092	0.018*	
C11A	0.40332 (6)	0.72029 (7)	0.05960 (4)	0.0129 (2)	
C12A	0.42293 (6)	0.64826 (6)	−0.02089 (4)	0.0127 (2)	
C13A	0.47480 (7)	0.61050 (6)	−0.00969 (4)	0.0133 (2)	
C14A	0.53007 (7)	0.59015 (7)	−0.03387 (4)	0.0156 (3)	
H14A	0.5414	0.5439	−0.0285	0.019*	
H14B	0.5690	0.6173	−0.0281	0.019*	
C15A	0.51536 (7)	0.59647 (7)	−0.07442 (4)	0.0142 (2)	
C16A	0.48509 (7)	0.66420 (7)	−0.08030 (4)	0.0158 (3)	
H16A	0.5192	0.6979	−0.0760	0.019*	
H16B	0.4710	0.6679	−0.1059	0.019*	
C17A	0.42705 (7)	0.67870 (7)	−0.05621 (4)	0.0141 (2)	
C18A	0.57947 (7)	0.59155 (7)	−0.09582 (4)	0.0187 (3)	
H18A	0.6093	0.6266	−0.0882	0.028*	
H18B	0.5702	0.5960	−0.1218	0.028*	
H18C	0.6000	0.5489	−0.0912	0.028*	
C19A	0.46737 (7)	0.54295 (7)	−0.08661 (4)	0.0192 (3)	
H19A	0.4258	0.5480	−0.0736	0.029*	
H19B	0.4861	0.4997	−0.0813	0.029*	
H19C	0.4595	0.5470	−0.1127	0.029*	
C20A	0.28941 (7)	0.80673 (8)	0.09259 (4)	0.0198 (3)	
H20A	0.2775	0.7656	0.0805	0.030*	
H20B	0.2553	0.8397	0.0881	0.030*	
H20C	0.2934	0.7991	0.1187	0.030*	
C21A	0.37389 (8)	0.89450 (7)	0.09753 (4)	0.0215 (3)	
H21A	0.3757	0.8862	0.1237	0.032*	
H21B	0.3412	0.9286	0.0925	0.032*	
H21C	0.4170	0.9093	0.0891	0.032*	
C4B	0.81079 (7)	0.38819 (7)	0.03626 (4)	0.0176 (3)	
C1B	0.8193 (3)	0.4552 (2)	0.03293 (13)	0.0218 (9)	0.735 (3)
H1BA	0.8443	0.4758	0.0144	0.026*	0.735 (3)
S1B	0.77821 (9)	0.49656 (7)	0.06707 (4)	0.0299 (3)	0.735 (3)
C2B	0.7426 (5)	0.4251 (5)	0.0842 (3)	0.0313 (18)	0.735 (3)
H2BA	0.7117	0.4229	0.1034	0.038*	0.735 (3)
C3B	0.7670 (7)	0.3731 (3)	0.0652 (3)	0.0218 (8)	0.735 (3)
H3BA	0.7553	0.3292	0.0710	0.026*	0.735 (3)
C1Y	0.7673 (19)	0.3684 (9)	0.0634 (10)	0.0218 (9)	0.265 (3)
H1YA	0.7542	0.3246	0.0683	0.026*	0.265 (3)
S1Y	0.7417 (4)	0.4372 (4)	0.0863 (2)	0.0358 (12)	0.265 (3)
C2Y	0.7818 (10)	0.4925 (8)	0.0583 (4)	0.037 (4)	0.265 (3)
H2YA	0.7763	0.5388	0.0574	0.044*	0.265 (3)
C3Y	0.8224 (9)	0.4555 (8)	0.0380 (5)	0.0218 (8)	0.265 (3)
H3YA	0.8581	0.4747	0.0253	0.026*	0.265 (3)
O1B	0.84132 (5)	0.30773 (6)	−0.06499 (3)	0.0207 (2)	
O2B	0.96238 (5)	0.40881 (5)	0.02813 (3)	0.0183 (2)	
O3B	0.81736 (6)	0.20770 (6)	−0.02075 (3)	0.0217 (2)	
O4B	0.91887 (5)	0.32452 (5)	0.07410 (3)	0.0181 (2)	
C5B	0.83837 (7)	0.33758 (7)	0.00993 (4)	0.0146 (2)	
H5BA	0.8003	0.3258	−0.0059	0.018*	
C6B	0.88949 (7)	0.36484 (7)	−0.01621 (4)	0.0148 (2)	
C7B	0.94425 (7)	0.39922 (7)	−0.00606 (4)	0.0149 (2)	
C8B	0.98958 (7)	0.43230 (7)	−0.03252 (4)	0.0179 (3)	
H8BA	1.0345	0.4324	−0.0223	0.021*	
H8BB	0.9757	0.4786	−0.0354	0.021*	
C9B	0.99160 (7)	0.39999 (7)	−0.06994 (4)	0.0180 (3)	
C10B	0.92029 (8)	0.38833 (8)	−0.08188 (4)	0.0207 (3)	
H10C	0.8989	0.4313	−0.0861	0.025*	
H10D	0.9203	0.3640	−0.1051	0.025*	
C11B	0.88085 (7)	0.35024 (7)	−0.05431 (4)	0.0168 (3)	
C12B	0.85772 (7)	0.27305 (7)	0.02810 (4)	0.0147 (2)	
C13B	0.84358 (7)	0.21397 (7)	0.01210 (4)	0.0168 (3)	
C14B	0.85774 (8)	0.14916 (7)	0.02953 (4)	0.0211 (3)	
H14C	0.8991	0.1316	0.0193	0.025*	
H14D	0.8221	0.1181	0.0232	0.025*	
C15B	0.86403 (7)	0.15195 (7)	0.07086 (4)	0.0181 (3)	
C16B	0.90965 (7)	0.20923 (7)	0.08039 (4)	0.0192 (3)	
H16C	0.9072	0.2169	0.1068	0.023*	
H16D	0.9553	0.1962	0.0747	0.023*	
C17B	0.89507 (7)	0.27293 (7)	0.06126 (4)	0.0150 (2)	
C18B	0.89383 (9)	0.08794 (8)	0.08484 (5)	0.0261 (3)	
H18D	0.8993	0.0906	0.1112	0.039*	
H18E	0.9367	0.0809	0.0734	0.039*	
H18F	0.8646	0.0513	0.0789	0.039*	
C19B	0.79651 (8)	0.16165 (8)	0.08853 (5)	0.0246 (3)	
H19D	0.8014	0.1623	0.1149	0.037*	
H19E	0.7674	0.1255	0.0816	0.037*	
H19F	0.7776	0.2033	0.0804	0.037*	
C20B	1.02941 (9)	0.33507 (8)	−0.06795 (5)	0.0274 (3)	
H20D	1.0754	0.3439	−0.0618	0.041*	
H20E	1.0272	0.3128	−0.0915	0.041*	
H20F	1.0099	0.3070	−0.0493	0.041*	
C21B	1.02532 (9)	0.44528 (8)	−0.09711 (4)	0.0243 (3)	
H21D	1.0703	0.4541	−0.0891	0.036*	
H21E	1.0010	0.4867	−0.0988	0.036*	
H21F	1.0263	0.4241	−0.1210	0.036*	
H1A3	0.4580 (12)	0.6109 (13)	0.0393 (7)	0.047 (7)*	
H1B2	0.9381 (13)	0.3845 (13)	0.0434 (7)	0.048 (7)*	
H1B3	0.8215 (12)	0.2413 (13)	−0.0329 (7)	0.044 (7)*	
H1A2	0.3405 (12)	0.7665 (13)	−0.0342 (7)	0.046 (7)*	

### Atomic displacement parameters (Å^2^)

**Table:**

	*U*^11^	*U*^22^	*U*^33^	*U*^12^	*U*^13^	*U*^23^
S1A	0.0251 (2)	0.0266 (2)	0.0283 (2)	−0.00512 (15)	0.00349 (15)	0.00306 (15)
O1A	0.0161 (5)	0.0160 (5)	0.0152 (5)	0.0019 (4)	−0.0022 (4)	0.0011 (4)
O2A	0.0244 (5)	0.0190 (5)	0.0120 (5)	0.0062 (4)	−0.0003 (4)	0.0023 (4)
O3A	0.0176 (5)	0.0181 (5)	0.0123 (4)	0.0031 (4)	−0.0013 (4)	0.0014 (4)
O4A	0.0201 (5)	0.0188 (5)	0.0141 (5)	0.0038 (4)	−0.0010 (4)	0.0022 (4)
C1A	0.0171 (6)	0.0165 (6)	0.0186 (6)	−0.0024 (5)	0.0017 (5)	0.0015 (5)
C2A	0.0150 (6)	0.0094 (5)	0.0165 (6)	0.0033 (4)	−0.0049 (5)	−0.0030 (4)
C3A	0.0180 (6)	0.0172 (6)	0.0199 (7)	−0.0029 (5)	0.0010 (5)	−0.0010 (5)
C4A	0.0118 (6)	0.0152 (6)	0.0150 (6)	−0.0012 (5)	−0.0022 (5)	0.0015 (5)
C5A	0.0121 (6)	0.0137 (6)	0.0121 (6)	0.0000 (4)	−0.0018 (4)	0.0009 (4)
C6A	0.0119 (6)	0.0137 (6)	0.0122 (6)	−0.0002 (4)	0.0004 (4)	0.0010 (4)
C7A	0.0140 (6)	0.0168 (6)	0.0121 (6)	0.0009 (5)	0.0016 (5)	0.0015 (5)
C8A	0.0218 (7)	0.0138 (6)	0.0148 (6)	0.0019 (5)	0.0035 (5)	0.0018 (5)
C9A	0.0146 (6)	0.0135 (6)	0.0137 (6)	−0.0001 (5)	0.0022 (5)	0.0005 (5)
C10A	0.0143 (6)	0.0152 (6)	0.0166 (6)	−0.0002 (5)	−0.0013 (5)	−0.0019 (5)
C11A	0.0108 (5)	0.0147 (6)	0.0133 (6)	−0.0012 (4)	0.0010 (4)	0.0009 (5)
C12A	0.0134 (6)	0.0123 (6)	0.0124 (6)	−0.0008 (4)	−0.0009 (4)	−0.0005 (4)
C13A	0.0157 (6)	0.0120 (6)	0.0123 (6)	−0.0014 (5)	−0.0011 (5)	−0.0005 (4)
C14A	0.0149 (6)	0.0164 (6)	0.0154 (6)	0.0026 (5)	−0.0005 (5)	−0.0013 (5)
C15A	0.0157 (6)	0.0126 (6)	0.0144 (6)	−0.0008 (5)	0.0006 (5)	−0.0017 (5)
C16A	0.0186 (6)	0.0148 (6)	0.0140 (6)	0.0003 (5)	0.0018 (5)	0.0013 (5)
C17A	0.0162 (6)	0.0137 (6)	0.0124 (6)	−0.0013 (5)	−0.0012 (5)	−0.0014 (5)
C18A	0.0190 (7)	0.0182 (7)	0.0188 (7)	−0.0007 (5)	0.0039 (5)	−0.0033 (5)
C19A	0.0205 (7)	0.0173 (6)	0.0198 (7)	−0.0038 (5)	0.0012 (5)	−0.0055 (5)
C20A	0.0171 (6)	0.0236 (7)	0.0189 (7)	−0.0009 (5)	0.0060 (5)	0.0012 (5)
C21A	0.0269 (8)	0.0159 (6)	0.0216 (7)	0.0000 (6)	−0.0003 (6)	−0.0039 (5)
C4B	0.0150 (6)	0.0233 (7)	0.0145 (6)	0.0062 (5)	−0.0038 (5)	−0.0030 (5)
C1B	0.0236 (13)	0.0194 (11)	0.022 (2)	0.0077 (10)	−0.0049 (14)	−0.0066 (12)
S1B	0.0293 (4)	0.0286 (4)	0.0317 (8)	0.0114 (3)	−0.0019 (5)	−0.0124 (4)
C2B	0.027 (3)	0.042 (4)	0.025 (3)	−0.002 (3)	−0.0004 (17)	0.005 (3)
C3B	0.0186 (11)	0.032 (2)	0.0153 (17)	0.0046 (17)	0.0008 (11)	−0.0021 (15)
C1Y	0.0236 (13)	0.0194 (11)	0.022 (2)	0.0077 (10)	−0.0049 (14)	−0.0066 (12)
S1Y	0.0376 (19)	0.045 (3)	0.0253 (14)	0.0207 (17)	−0.0076 (11)	−0.0104 (14)
C2Y	0.041 (6)	0.051 (6)	0.018 (6)	0.015 (5)	−0.015 (4)	−0.005 (4)
C3Y	0.0186 (11)	0.032 (2)	0.0153 (17)	0.0046 (17)	0.0008 (11)	−0.0021 (15)
O1B	0.0212 (5)	0.0246 (5)	0.0163 (5)	−0.0045 (4)	−0.0028 (4)	−0.0036 (4)
O2B	0.0219 (5)	0.0187 (5)	0.0143 (5)	−0.0043 (4)	−0.0039 (4)	0.0000 (4)
O3B	0.0253 (6)	0.0230 (6)	0.0167 (5)	−0.0058 (4)	−0.0008 (4)	−0.0039 (4)
O4B	0.0190 (5)	0.0184 (5)	0.0169 (5)	−0.0016 (4)	−0.0034 (4)	−0.0001 (4)
C5B	0.0134 (6)	0.0172 (6)	0.0132 (6)	0.0015 (5)	−0.0022 (5)	−0.0021 (5)
C6B	0.0164 (6)	0.0150 (6)	0.0130 (6)	0.0012 (5)	−0.0012 (5)	−0.0006 (5)
C7B	0.0189 (6)	0.0118 (6)	0.0140 (6)	0.0018 (5)	−0.0021 (5)	−0.0006 (5)
C8B	0.0213 (7)	0.0149 (6)	0.0175 (6)	−0.0039 (5)	−0.0008 (5)	0.0017 (5)
C9B	0.0211 (7)	0.0136 (6)	0.0193 (7)	−0.0006 (5)	0.0033 (5)	0.0000 (5)
C10B	0.0254 (7)	0.0223 (7)	0.0143 (6)	−0.0026 (6)	0.0005 (5)	−0.0011 (5)
C11B	0.0174 (6)	0.0179 (6)	0.0152 (6)	0.0008 (5)	−0.0011 (5)	−0.0018 (5)
C12B	0.0132 (6)	0.0164 (6)	0.0144 (6)	0.0003 (5)	0.0011 (5)	−0.0009 (5)
C13B	0.0144 (6)	0.0198 (7)	0.0163 (6)	−0.0025 (5)	0.0030 (5)	−0.0020 (5)
C14B	0.0263 (7)	0.0159 (6)	0.0211 (7)	−0.0021 (5)	0.0053 (6)	−0.0022 (5)
C15B	0.0180 (6)	0.0163 (6)	0.0200 (7)	−0.0006 (5)	0.0056 (5)	0.0005 (5)
C16B	0.0184 (7)	0.0186 (7)	0.0208 (7)	0.0002 (5)	−0.0011 (5)	0.0039 (5)
C17B	0.0124 (6)	0.0174 (6)	0.0152 (6)	0.0012 (5)	0.0014 (5)	0.0004 (5)
C18B	0.0305 (8)	0.0188 (7)	0.0289 (8)	0.0031 (6)	0.0073 (7)	0.0054 (6)
C19B	0.0214 (7)	0.0266 (8)	0.0257 (8)	0.0002 (6)	0.0088 (6)	0.0014 (6)
C20B	0.0284 (8)	0.0173 (7)	0.0364 (9)	0.0032 (6)	0.0103 (7)	0.0020 (6)
C21B	0.0308 (8)	0.0209 (7)	0.0212 (7)	−0.0041 (6)	0.0063 (6)	0.0020 (6)

### Geometric parameters (Å, °)

**Table:**

S1A—C2A	1.6829 (14)	C4B—C5B	1.5244 (19)
S1A—C1A	1.7071 (15)	C1B—S1B	1.732 (6)
O1A—C11A	1.2530 (16)	C1B—H1BA	0.9500
O2A—C7A	1.3368 (16)	S1B—C2B	1.745 (9)
O2A—H1A2	0.87 (3)	C2B—C3B	1.364 (11)
O3A—C13A	1.3308 (16)	C2B—H2BA	0.9500
O3A—H1A3	0.88 (3)	C3B—H3BA	0.9500
O4A—C17A	1.2524 (17)	C1Y—S1Y	1.718 (19)
C1A—C4A	1.3722 (19)	C1Y—H1YA	0.9500
C1A—H1AA	0.9500	S1Y—C2Y	1.734 (14)
C2A—C3A	1.458 (2)	C2Y—C3Y	1.346 (19)
C2A—H2AA	0.9500	C2Y—H2YA	0.9500
C3A—C4A	1.4246 (19)	C3Y—H3YA	0.9500
C3A—H3AA	0.9500	O1B—C11B	1.2467 (18)
C4A—C5A	1.5249 (18)	O2B—C7B	1.3291 (16)
C5A—C6A	1.5247 (18)	O2B—H1B2	0.90 (3)
C5A—C12A	1.5252 (18)	O3B—C13B	1.3309 (18)
C5A—H5AA	1.0000	O3B—H1B3	0.82 (3)
C6A—C7A	1.3708 (18)	O4B—C17B	1.2510 (17)
C6A—C11A	1.4452 (18)	C5B—C6B	1.5245 (19)
C7A—C8A	1.4995 (19)	C5B—C12B	1.5278 (19)
C8A—C9A	1.5323 (19)	C5B—H5BA	1.0000
C8A—H8AA	0.9900	C6B—C7B	1.3699 (19)
C8A—H8AB	0.9900	C6B—C11B	1.4481 (19)
C9A—C20A	1.5265 (19)	C7B—C8B	1.504 (2)
C9A—C21A	1.5293 (19)	C8B—C9B	1.531 (2)
C9A—C10A	1.5293 (19)	C8B—H8BA	0.9900
C10A—C11A	1.5069 (19)	C8B—H8BB	0.9900
C10A—H10A	0.9900	C9B—C21B	1.526 (2)
C10A—H10B	0.9900	C9B—C20B	1.533 (2)
C12A—C13A	1.3711 (18)	C9B—C10B	1.537 (2)
C12A—C17A	1.4459 (18)	C10B—C11B	1.511 (2)
C13A—C14A	1.4956 (19)	C10B—H10C	0.9900
C14A—C15A	1.5317 (19)	C10B—H10D	0.9900
C14A—H14A	0.9900	C12B—C13B	1.3715 (19)
C14A—H14B	0.9900	C12B—C17B	1.4414 (19)
C15A—C16A	1.5272 (19)	C13B—C14B	1.497 (2)
C15A—C18A	1.5298 (19)	C14B—C15B	1.532 (2)
C15A—C19A	1.5324 (19)	C14B—H14C	0.9900
C16A—C17A	1.5087 (19)	C14B—H14D	0.9900
C16A—H16A	0.9900	C15B—C18B	1.528 (2)
C16A—H16B	0.9900	C15B—C16B	1.533 (2)
C18A—H18A	0.9800	C15B—C19B	1.535 (2)
C18A—H18B	0.9800	C16B—C17B	1.507 (2)
C18A—H18C	0.9800	C16B—H16C	0.9900
C19A—H19A	0.9800	C16B—H16D	0.9900
C19A—H19B	0.9800	C18B—H18D	0.9800
C19A—H19C	0.9800	C18B—H18E	0.9800
C20A—H20A	0.9800	C18B—H18F	0.9800
C20A—H20B	0.9800	C19B—H19D	0.9800
C20A—H20C	0.9800	C19B—H19E	0.9800
C21A—H21A	0.9800	C19B—H19F	0.9800
C21A—H21B	0.9800	C20B—H20D	0.9800
C21A—H21C	0.9800	C20B—H20E	0.9800
C4B—C1B	1.382 (5)	C20B—H20F	0.9800
C4B—C3Y	1.393 (16)	C21B—H21D	0.9800
C4B—C1Y	1.396 (18)	C21B—H21E	0.9800
C4B—C3B	1.426 (6)	C21B—H21F	0.9800
			
C2A—S1A—C1A	94.78 (7)	C4B—C1B—S1B	110.8 (3)
C7A—O2A—H1A2	109.6 (17)	C4B—C1B—H1BA	124.6
C13A—O3A—H1A3	111.2 (17)	S1B—C1B—H1BA	124.6
C4A—C1A—S1A	112.43 (11)	C1B—S1B—C2B	93.4 (4)
C4A—C1A—H1AA	123.8	C3B—C2B—S1B	108.1 (6)
S1A—C1A—H1AA	123.8	C3B—C2B—H2BA	125.9
C3A—C2A—S1A	108.42 (10)	S1B—C2B—H2BA	125.9
C3A—C2A—H2AA	125.8	C2B—C3B—C4B	116.4 (6)
S1A—C2A—H2AA	125.8	C2B—C3B—H3BA	121.8
C4A—C3A—C2A	113.14 (12)	C4B—C3B—H3BA	121.8
C4A—C3A—H3AA	123.4	C4B—C1Y—S1Y	108.1 (11)
C2A—C3A—H3AA	123.4	C4B—C1Y—H1YA	126.0
C1A—C4A—C3A	111.21 (12)	S1Y—C1Y—H1YA	126.0
C1A—C4A—C5A	124.37 (12)	C1Y—S1Y—C2Y	95.3 (9)
C3A—C4A—C5A	124.13 (12)	C3Y—C2Y—S1Y	104.9 (12)
C6A—C5A—C4A	113.88 (11)	C3Y—C2Y—H2YA	127.5
C6A—C5A—C12A	113.57 (11)	S1Y—C2Y—H2YA	127.5
C4A—C5A—C12A	114.35 (11)	C2Y—C3Y—C4B	118.2 (14)
C6A—C5A—H5AA	104.5	C2Y—C3Y—H3YA	120.9
C4A—C5A—H5AA	104.5	C4B—C3Y—H3YA	120.9
C12A—C5A—H5AA	104.5	C7B—O2B—H1B2	111.2 (16)
C7A—C6A—C11A	117.78 (12)	C13B—O3B—H1B3	112.0 (18)
C7A—C6A—C5A	121.60 (12)	C4B—C5B—C6B	114.13 (12)
C11A—C6A—C5A	120.37 (11)	C4B—C5B—C12B	113.43 (11)
O2A—C7A—C6A	123.70 (13)	C6B—C5B—C12B	114.51 (11)
O2A—C7A—C8A	112.39 (12)	C4B—C5B—H5BA	104.4
C6A—C7A—C8A	123.89 (12)	C6B—C5B—H5BA	104.4
C7A—C8A—C9A	114.25 (11)	C12B—C5B—H5BA	104.4
C7A—C8A—H8AA	108.7	C7B—C6B—C11B	118.01 (13)
C9A—C8A—H8AA	108.7	C7B—C6B—C5B	124.75 (12)
C7A—C8A—H8AB	108.7	C11B—C6B—C5B	117.17 (12)
C9A—C8A—H8AB	108.7	O2B—C7B—C6B	124.14 (13)
H8AA—C8A—H8AB	107.6	O2B—C7B—C8B	112.32 (12)
C20A—C9A—C21A	109.09 (12)	C6B—C7B—C8B	123.51 (12)
C20A—C9A—C10A	110.41 (11)	C7B—C8B—C9B	114.18 (12)
C21A—C9A—C10A	109.16 (12)	C7B—C8B—H8BA	108.7
C20A—C9A—C8A	110.60 (12)	C9B—C8B—H8BA	108.7
C21A—C9A—C8A	109.41 (11)	C7B—C8B—H8BB	108.7
C10A—C9A—C8A	108.13 (11)	C9B—C8B—H8BB	108.7
C11A—C10A—C9A	114.85 (11)	H8BA—C8B—H8BB	107.6
C11A—C10A—H10A	108.6	C21B—C9B—C8B	110.18 (12)
C9A—C10A—H10A	108.6	C21B—C9B—C20B	109.09 (13)
C11A—C10A—H10B	108.6	C8B—C9B—C20B	109.97 (13)
C9A—C10A—H10B	108.6	C21B—C9B—C10B	109.31 (13)
H10A—C10A—H10B	107.5	C8B—C9B—C10B	107.45 (12)
O1A—C11A—C6A	121.57 (12)	C20B—C9B—C10B	110.83 (13)
O1A—C11A—C10A	118.08 (12)	C11B—C10B—C9B	112.90 (12)
C6A—C11A—C10A	120.27 (12)	C11B—C10B—H10C	109.0
C13A—C12A—C17A	117.89 (12)	C9B—C10B—H10C	109.0
C13A—C12A—C5A	125.17 (12)	C11B—C10B—H10D	109.0
C17A—C12A—C5A	116.88 (11)	C9B—C10B—H10D	109.0
O3A—C13A—C12A	123.70 (12)	H10C—C10B—H10D	107.8
O3A—C13A—C14A	112.51 (12)	O1B—C11B—C6B	121.90 (13)
C12A—C13A—C14A	123.79 (12)	O1B—C11B—C10B	119.18 (13)
C13A—C14A—C15A	114.35 (11)	C6B—C11B—C10B	118.92 (12)
C13A—C14A—H14A	108.7	C13B—C12B—C17B	118.36 (13)
C15A—C14A—H14A	108.7	C13B—C12B—C5B	120.82 (12)
C13A—C14A—H14B	108.7	C17B—C12B—C5B	120.69 (12)
C15A—C14A—H14B	108.7	O3B—C13B—C12B	124.10 (14)
H14A—C14A—H14B	107.6	O3B—C13B—C14B	112.59 (13)
C16A—C15A—C18A	109.32 (11)	C12B—C13B—C14B	123.29 (13)
C16A—C15A—C14A	107.09 (11)	C13B—C14B—C15B	114.29 (12)
C18A—C15A—C14A	109.38 (11)	C13B—C14B—H14C	108.7
C16A—C15A—C19A	110.11 (12)	C15B—C14B—H14C	108.7
C18A—C15A—C19A	110.28 (11)	C13B—C14B—H14D	108.7
C14A—C15A—C19A	110.60 (12)	C15B—C14B—H14D	108.7
C17A—C16A—C15A	114.20 (11)	H14C—C14B—H14D	107.6
C17A—C16A—H16A	108.7	C18B—C15B—C14B	109.73 (13)
C15A—C16A—H16A	108.7	C18B—C15B—C16B	109.35 (13)
C17A—C16A—H16B	108.7	C14B—C15B—C16B	107.90 (12)
C15A—C16A—H16B	108.7	C18B—C15B—C19B	108.82 (12)
H16A—C16A—H16B	107.6	C14B—C15B—C19B	110.66 (13)
O4A—C17A—C12A	121.85 (12)	C16B—C15B—C19B	110.36 (12)
O4A—C17A—C16A	118.57 (12)	C17B—C16B—C15B	115.39 (12)
C12A—C17A—C16A	119.51 (12)	C17B—C16B—H16C	108.4
C15A—C18A—H18A	109.5	C15B—C16B—H16C	108.4
C15A—C18A—H18B	109.5	C17B—C16B—H16D	108.4
H18A—C18A—H18B	109.5	C15B—C16B—H16D	108.4
C15A—C18A—H18C	109.5	H16C—C16B—H16D	107.5
H18A—C18A—H18C	109.5	O4B—C17B—C12B	121.67 (13)
H18B—C18A—H18C	109.5	O4B—C17B—C16B	118.02 (12)
C15A—C19A—H19A	109.5	C12B—C17B—C16B	120.22 (12)
C15A—C19A—H19B	109.5	C15B—C18B—H18D	109.5
H19A—C19A—H19B	109.5	C15B—C18B—H18E	109.5
C15A—C19A—H19C	109.5	H18D—C18B—H18E	109.5
H19A—C19A—H19C	109.5	C15B—C18B—H18F	109.5
H19B—C19A—H19C	109.5	H18D—C18B—H18F	109.5
C9A—C20A—H20A	109.5	H18E—C18B—H18F	109.5
C9A—C20A—H20B	109.5	C15B—C19B—H19D	109.5
H20A—C20A—H20B	109.5	C15B—C19B—H19E	109.5
C9A—C20A—H20C	109.5	H19D—C19B—H19E	109.5
H20A—C20A—H20C	109.5	C15B—C19B—H19F	109.5
H20B—C20A—H20C	109.5	H19D—C19B—H19F	109.5
C9A—C21A—H21A	109.5	H19E—C19B—H19F	109.5
C9A—C21A—H21B	109.5	C9B—C20B—H20D	109.5
H21A—C21A—H21B	109.5	C9B—C20B—H20E	109.5
C9A—C21A—H21C	109.5	H20D—C20B—H20E	109.5
H21A—C21A—H21C	109.5	C9B—C20B—H20F	109.5
H21B—C21A—H21C	109.5	H20D—C20B—H20F	109.5
C1B—C4B—C1Y	115.4 (9)	H20E—C20B—H20F	109.5
C3Y—C4B—C1Y	111.0 (8)	C9B—C21B—H21D	109.5
C1B—C4B—C3B	111.0 (3)	C9B—C21B—H21E	109.5
C3Y—C4B—C3B	106.5 (9)	H21D—C21B—H21E	109.5
C1B—C4B—C5B	124.4 (3)	C9B—C21B—H21F	109.5
C3Y—C4B—C5B	129.2 (7)	H21D—C21B—H21F	109.5
C1Y—C4B—C5B	119.7 (8)	H21E—C21B—H21F	109.5
C3B—C4B—C5B	124.2 (3)		
			
C2A—S1A—C1A—C4A	−1.00 (12)	C5B—C4B—C3B—C2B	−174.5 (9)
C1A—S1A—C2A—C3A	0.84 (11)	C1B—C4B—C1Y—S1Y	−4(4)
S1A—C2A—C3A—C4A	−0.53 (15)	C3Y—C4B—C1Y—S1Y	4(3)
S1A—C1A—C4A—C3A	0.82 (16)	C3B—C4B—C1Y—S1Y	14 (32)
S1A—C1A—C4A—C5A	174.82 (10)	C5B—C4B—C1Y—S1Y	−176.2 (13)
C2A—C3A—C4A—C1A	−0.19 (17)	C4B—C1Y—S1Y—C2Y	5(3)
C2A—C3A—C4A—C5A	−174.20 (12)	C1Y—S1Y—C2Y—C3Y	−12 (3)
C1A—C4A—C5A—C6A	24.20 (18)	S1Y—C2Y—C3Y—C4B	17 (2)
C3A—C4A—C5A—C6A	−162.57 (13)	C1B—C4B—C3Y—C2Y	109 (7)
C1A—C4A—C5A—C12A	157.06 (13)	C1Y—C4B—C3Y—C2Y	−15 (2)
C3A—C4A—C5A—C12A	−29.70 (18)	C3B—C4B—C3Y—C2Y	−15 (2)
C4A—C5A—C6A—C7A	−133.08 (13)	C5B—C4B—C3Y—C2Y	165.4 (12)
C12A—C5A—C6A—C7A	93.68 (15)	C1B—C4B—C5B—C6B	13.1 (3)
C4A—C5A—C6A—C11A	52.79 (16)	C3Y—C4B—C5B—C6B	4.8 (10)
C12A—C5A—C6A—C11A	−80.45 (15)	C1Y—C4B—C5B—C6B	−175 (2)
C11A—C6A—C7A—O2A	168.04 (12)	C3B—C4B—C5B—C6B	−174.1 (8)
C5A—C6A—C7A—O2A	−6.2 (2)	C1B—C4B—C5B—C12B	146.7 (3)
C11A—C6A—C7A—C8A	−10.3 (2)	C3Y—C4B—C5B—C12B	138.4 (10)
C5A—C6A—C7A—C8A	175.43 (12)	C1Y—C4B—C5B—C12B	−42 (2)
O2A—C7A—C8A—C9A	163.14 (12)	C3B—C4B—C5B—C12B	−40.5 (9)
C6A—C7A—C8A—C9A	−18.4 (2)	C4B—C5B—C6B—C7B	52.84 (18)
C7A—C8A—C9A—C20A	−74.73 (15)	C12B—C5B—C6B—C7B	−80.25 (17)
C7A—C8A—C9A—C21A	165.07 (12)	C4B—C5B—C6B—C11B	−130.44 (13)
C7A—C8A—C9A—C10A	46.28 (16)	C12B—C5B—C6B—C11B	96.47 (15)
C20A—C9A—C10A—C11A	72.02 (15)	C11B—C6B—C7B—O2B	−171.28 (13)
C21A—C9A—C10A—C11A	−168.06 (12)	C5B—C6B—C7B—O2B	5.4 (2)
C8A—C9A—C10A—C11A	−49.11 (15)	C11B—C6B—C7B—C8B	10.8 (2)
C7A—C6A—C11A—O1A	−169.28 (12)	C5B—C6B—C7B—C8B	−172.53 (13)
C5A—C6A—C11A—O1A	5.06 (19)	O2B—C7B—C8B—C9B	153.75 (12)
C7A—C6A—C11A—C10A	7.43 (19)	C6B—C7B—C8B—C9B	−28.1 (2)
C5A—C6A—C11A—C10A	−178.22 (12)	C7B—C8B—C9B—C21B	166.61 (13)
C9A—C10A—C11A—O1A	−159.26 (12)	C7B—C8B—C9B—C20B	−73.10 (16)
C9A—C10A—C11A—C6A	23.91 (18)	C7B—C8B—C9B—C10B	47.61 (16)
C6A—C5A—C12A—C13A	88.43 (16)	C21B—C9B—C10B—C11B	−173.48 (12)
C4A—C5A—C12A—C13A	−44.59 (18)	C8B—C9B—C10B—C11B	−53.92 (16)
C6A—C5A—C12A—C17A	−88.90 (14)	C20B—C9B—C10B—C11B	66.25 (16)
C4A—C5A—C12A—C17A	138.08 (12)	C7B—C6B—C11B—O1B	163.83 (14)
C17A—C12A—C13A—O3A	169.83 (12)	C5B—C6B—C11B—O1B	−13.1 (2)
C5A—C12A—C13A—O3A	−7.5 (2)	C7B—C6B—C11B—C10B	−17.0 (2)
C17A—C12A—C13A—C14A	−10.3 (2)	C5B—C6B—C11B—C10B	166.05 (13)
C5A—C12A—C13A—C14A	172.44 (12)	C9B—C10B—C11B—O1B	−140.33 (14)
O3A—C13A—C14A—C15A	162.39 (11)	C9B—C10B—C11B—C6B	40.48 (18)
C12A—C13A—C14A—C15A	−17.53 (19)	C4B—C5B—C12B—C13B	138.17 (13)
C13A—C14A—C15A—C16A	47.25 (15)	C6B—C5B—C12B—C13B	−88.41 (16)
C13A—C14A—C15A—C18A	165.61 (12)	C4B—C5B—C12B—C17B	−45.95 (17)
C13A—C14A—C15A—C19A	−72.74 (15)	C6B—C5B—C12B—C17B	87.47 (15)
C18A—C15A—C16A—C17A	−171.28 (11)	C17B—C12B—C13B—O3B	−170.66 (13)
C14A—C15A—C16A—C17A	−52.88 (15)	C5B—C12B—C13B—O3B	5.3 (2)
C19A—C15A—C16A—C17A	67.43 (15)	C17B—C12B—C13B—C14B	7.7 (2)
C13A—C12A—C17A—O4A	−172.58 (13)	C5B—C12B—C13B—C14B	−176.31 (13)
C5A—C12A—C17A—O4A	4.95 (19)	O3B—C13B—C14B—C15B	−159.09 (13)
C13A—C12A—C17A—C16A	4.36 (19)	C12B—C13B—C14B—C15B	22.4 (2)
C5A—C12A—C17A—C16A	−178.12 (11)	C13B—C14B—C15B—C18B	−166.86 (13)
C15A—C16A—C17A—O4A	−153.95 (12)	C13B—C14B—C15B—C16B	−47.79 (17)
C15A—C16A—C17A—C12A	29.01 (17)	C13B—C14B—C15B—C19B	73.04 (16)
C3Y—C4B—C1B—S1B	−54 (7)	C18B—C15B—C16B—C17B	166.62 (13)
C1Y—C4B—C1B—S1B	6(3)	C14B—C15B—C16B—C17B	47.31 (17)
C3B—C4B—C1B—S1B	4.1 (7)	C19B—C15B—C16B—C17B	−73.70 (16)
C5B—C4B—C1B—S1B	177.77 (17)	C13B—C12B—C17B—O4B	168.08 (13)
C4B—C1B—S1B—C2B	−5.0 (5)	C5B—C12B—C17B—O4B	−7.9 (2)
C1B—S1B—C2B—C3B	4.4 (11)	C13B—C12B—C17B—C16B	−8.5 (2)
S1B—C2B—C3B—C4B	−2.9 (17)	C5B—C12B—C17B—C16B	175.52 (12)
C1B—C4B—C3B—C2B	−0.8 (15)	C15B—C16B—C17B—O4B	162.45 (13)
C3Y—C4B—C3B—C2B	6(2)	C15B—C16B—C17B—C12B	−20.85 (19)
C1Y—C4B—C3B—C2B	−163 (35)		

### Hydrogen-bond geometry (Å, °)

**Table:**

*D*—H···*A*	*D*—H	H···*A*	*D*···*A*	*D*—H···*A*
O3A—H1A3···O1A	0.88 (3)	1.71 (3)	2.5638 (15)	164 (2)
O2B—H1B2···O4B	0.90 (3)	1.71 (3)	2.5719 (15)	159 (3)
O3B—H1B3···O1B	0.82 (3)	1.84 (3)	2.6569 (17)	170 (3)
O2A—H1A2···O4A	0.87 (3)	1.79 (3)	2.6393 (15)	165 (2)
C10A—H10A···O1A^i^	0.99	2.48	3.4530 (18)	169
C16B—H16D···O4B^ii^	0.99	2.60	3.5688 (18)	167

Symmetry codes: (i) −*x*+1, −*y*+3/2, *z*; (ii) −*x*+2, −*y*+1/2, *z*.
